# Mathematical Approach for the Assessment of Similarity Factor Using a New Scheme for Calculating Weight

**DOI:** 10.4103/0250-474X.54281

**Published:** 2009

**Authors:** M. C. Gohel, K. G. Sarvaiya, A. R. Shah, B. K. Brahmbhatt

**Affiliations:** Department of Pharmaceutics and Pharmaceutical Technology, L. M. College of Pharmacy, Navrangpura, Ahmedabad-380 009, India

**Keywords:** Similarity factor, Weight, Dissolution, Coefficient of Variation

## Abstract

The objective of the present work was to propose a method for calculating weight in the Moore and Flanner Equation. The percentage coefficient of variation in reference and test formulations at each time point was considered for calculating weight. The literature reported data are used to demonstrate applicability of the method. The advantages and applications of new approach are narrated. The results show a drop in the value of similarity factor as compared to the approach proposed in earlier work. The scientists who need high accuracy in calculation may use this approach.

Moore and Flanner proposed two new indices (f_1_ and f_2_) to compare dissolution profiles of a test and a reference formulations[1]. The concept of similarity factor (f_2_) has been endorsed by Food and Drug Administration (FDA); therefore, it is widely adopted in formulation and development and dossier preparation. Earlier, we suggested three different schemes for calculating weight to compute similarity factor[2]. In the present work an additional scheme for calculating weight is proposed and its impact on value of similarity factor is compared with the best approach proposed in our earlier work[2]. The equation of similarity factor proposed by Moore and Flanner is represented in Eqn. 1[1],

(1) $$f_{2}=50\log\left\{{\left[1+\frac{1}{n}\sum_{n-1}^{n}{\left(R_{t}-T_{t}\right)}^{2}\right]}^{-0.5}*100\right\}$$

where, f_2_ is similarity factor, n is the number of observations, w_t_ is optional weight, R_t_ is average percentage drug dissolved from reference formulation and T_t_ is average percentage drug dissolved from test formulation. Generally, an average of twelve observations is used for calculating f_2_. The weight factor (w_t_) is usually considered as unity.

FDA states that in the instances where within batch variability is more than 15% coefficient of variation (CV), a multivariate model independent procedure is more suitable for dissolution profile comparison. It is further stated in the guidance document that to allow use of mean data, the percent coefficient of variation (%CV) should not be more than 20% at the earlier time points, and at the other time points %CV should not be more than 10%[3]. These values are considered as maximum allowable %CV in the present work. One of the objectives of the proposed work was to incorporate these conditions in calculation of f_2_. The new scheme for calculating weight shows impact of within sample variability on f_2_ value. Weight (w_t_) is calculated using Eqn. 2, w_t_ =1+{(%CVof R_t_)/(MCV_E/L_)}+{(%CV of T_t_)/(MCV_E/L_)}--(2), where, w_t_ is weight, %CV of R_t_ and %CV of T_t_ are the percentage coefficient of variation of reference and test products respectively. MCV_E/L_ is the maximum allowable %CV. MCV_E/L_ was 20 for earlier time point (30 min) and it was 10 for later time points (above 30 min). Co-efficient of variation was calculated using the Eqn. 3. If the %CV of R_t_ and %CV of T_t_ is equal to zero, w_t_ is equal to one. %CV=(standard deviation /mean)×100--(3).

In our earlier publication, we stated that the approach 3 was the best amongst other approaches for calculating weight. In approach 3, reference product (12 units) and test product (12 units) were used to generate 144 values of absolute differences between a reference and a test formulation at the four sampling time points (30, 60, 90 and 180 min). Standard deviation (SD) of the 144 values was calculated. The twelve units of test formulation will show different dissolution profiles and this variability is referred to as between samples variability. The weight was calculated from the Eqn.: (1+standard deviation/maximum allowed standard deviation). The maximum allowed standard deviation was arbitrarily chosen as 10 to allow within samples as well as variability between samples. In was arbitrarily decided to give weight equal to one when standard deviation is zero.

The value of similarity factor (f_2_) is 100 when the difference between reference and test formulation is zero and weight (w_t_) is unity. Previously, we reported that as the value of weight (w_t_) increases, a decrease in the value of similarity factor is anticipated[2]. In the present work, two ratio terms are included in Eqn. 2 to cause a drop in similarity factor as variability increases.

To demonstrate the utility of new weight approach, the dissolution data reported by Shah *et al.* were used[4]. The average value of cumulative percentage drug dissolved for reference (R) and (T) formulations are shown in Table 1. The standard deviation and average of dissolution data of reference and test formulations were calculated at each sampling time. The results are shown in Table 2.

**TABLE 1:** DISSOLUTION DATA FOR CALCULATING F_2_ VALUES[4]

Reference			Test 1 (n = 12)			Test 2 (n = 12)		
		
Time (min)	Average	SD	Time (min)	Average	SD	Time (min)	Average	SD
30	34.92	2.26	30	40.34	4.10	30	49.33	2.32
60	59.60	3.85	60	67.15	6.34	60	65.33	5.02
90	79.27	5.12	90	87.01	4.76	90	86.75	3.52
180	95.08	6.14	180	97.73	1.48	180	102.83	1.72
			f_2_ = 60.04			f_2_ = 51.08		

**Test 3 (n = 12)**			**Test 4 (n = 12)**			**Test 5 (n = 12)**		
		
**Time (min)**	**Average**	**SD**	**Time (min)**	**Average**	**SD**	**Time (min)**	**Average**	**SD**

30	25.80	2.36	30	15.08	5.78	30	43.39	1.29
60	50.64	4.46	60	59.50	3.07	60	77.96	1.43
90	67.00	6.14	90	79.27	4.32	90	86.33	2.80
180	88.60	8.12	180	95.08	2.68	180	95.58	1.99
	f_2_ = 51.19			f_2_ = 50.07			f_2_ = 48.05	

SD = standard deviation, f_2_ = similarity factor

**TABLE 2:** SAMPLE CALCULATION FOR WEIGHT (W_T_) FOR TEST FORMULATION1

Time (min)	R	T	SDR	SDT	CVR	CVT	w_t_
30	34.92	40.34	2.26	4.10	6.47	10.16	1.83
60	59.50	67.15	3.85	6.34	6.47	9.44	2.59
90	79.27	87.01	5.12	4.76	6.45	5.47	2.19
180	95.08	97.73	6.14	1.48	6.45	1.51	1.79

R = reference, T = Test, SDR = standard deviation of R_t_, SDT = standard deviation of T_t_, CVR = percentage coefficient of variation of R_t_, CVT = percentage coefficient variation of T_t_, w_t_ = 1 + (%CV of R_t_/MCV_E/L_) + (% CV of T_t_/MCVE/L)

Table 3 displays the value of similarity factor calculated using the new approach (f_2-m_) and approach 3 (f_2-m3_) of our earlier publication. The results show that the value of f_2-m_ was lower than the value of f_2-m3_ in all the cases (test 1 to test 5). The new approach appears to be more sensitive than the approach 3 proposed in our publication since within sample variability is incorporated in the new approach. If the average value of reference and test at all the time point is similar then it is irrational to compute weight for calculating similarity factor because the product of weight (w_t_) and (R_t_−T_t_) will be zero. Therefore f_2_ is equal to 100.

**TABLE 3:** SIMILARITY FACTORS FOR DIFFERENT TEST FORMULATIONS

Test formulations	f_2-m_	f_2-m3_	f_2_
1	51.34	54.68	60.04
2	45.01	46.88	51.08
3	41.86	48.30	51.19
4	37.38	46.46	50.07
5	42.05	44.98	48.05

f_2-m_ = Similarity factor calculated using new approach, f_2-m3_ = Similarity factor calculated using approach 3, f_2_ = Similarity factor calculated using conventional method (w_t_ = 1)

In the new scheme of weight (w_t_) calculation, no parameter was decided on an arbitrary ground. The new approach appears to be more realistic as compared to approach 3. Another advantage of the new method is simple calculation steps than approach 3. Equal stress is given to variability in reference and test formulation in the new approach. The maximum allowable %CV is also considered in the proposed method. It considers within samples (12 units of reference and 12 units of test) as well as between samples (reference and test formulations) variability because of utilization of standard deviation and averages of reference and test formulations at each time points for calculating weight. The use of new method is recommended in deciding equivalence of test product with innovators product. The approach may also find application in selection of a bio-batch. The use of new approach may become a strong point in Abbreviated New Drug Application (ANDA) submission. If the value of f_2-m_ is greater than 50 than we may safely conclude that products show similar dissolution. The positive and negative points of the new approach will emerge out when various researchers will try the approach with their data sets.
